# Nanoscale Size-Selective Deposition of Nanowires by Micrometer Scale Hydrophilic Patterns

**DOI:** 10.1038/srep05943

**Published:** 2014-08-04

**Authors:** Yong He, Kazuki Nagashima, Masaki Kanai, Gang Meng, Fuwei Zhuge, Sakon Rahong, Xiaomin Li, Tomoji Kawai, Takeshi Yanagida

**Affiliations:** 1 The Institute of Scientific and Industrial Research, Osaka University, 8-1 Mihogaoka Ibaraki, Osaka, 567-0047, Japan; 2 Shanghai Institute of Ceramics, Chinese Academy of Sciences, Shanghai, 200050, China

## Abstract

Controlling the post-growth assembly of nanowires is an important challenge in the development of functional bottom-up devices. Although various methods have been developed for the controlled assembly of nanowires, it is still a challenging issue to align selectively heterogeneous nanowires at desired spatial positions on the substrate. Here we report a size selective deposition and sequential alignment of nanowires by utilizing micrometer scale hydrophilic/hydrophobic patterned substrate. Nanowires dispersed within oil were preferentially deposited only at a water/oil interface onto the hydrophilic patterns. The diameter size of deposited nanowires was strongly limited by the width of hydrophilic patterns, exhibiting the nanoscale size selectivity of nanowires deposited onto micrometer scale hydrophilic patterns. Such size selectivity was due to the nanoscale height variation of a water layer formed onto the micrometer scale hydrophilic patterns. We successfully demonstrated the sequential alignment of different sized nanowires on the same substrate by applying this size selective phenomenon.

Designing the post-growth assembly of nanowires on the substrate offers a fascinating way to explore novel functional nanoscale devices. In general, such assembling processes of nanowires can be performed at relatively low temperatures^1^,^2^,^3^,^4^,^5^,^6^,^7^,^8^,^9^,^10^,^11^,^12^,^13^,^14^,^15^,^16^,^17^,^18^,^19^,^20^,^21^,^22^,^23^,^24^,^25^,^26^, which are much lower than typical nanowire growth temperatures^27^,^28^,^29^,^30^,^31^,^32^. Therefore, this assembling method allows us to integrate single crystalline nanowires, which are grown at relatively high temperatures, on various substrates via low temperature processes. This is a clear feature of the assembling method, since high temperature processes limit the range of substrate materials employed. Furthermore, this assembling technique has a potential to integrate heterogeneous single crystalline nanowires on the same substrate. Such integration of heterogeneous single crystalline objects on the same substrate is rather difficult to be accomplished via conventional growth and lithography techniques. Previous studies have demonstrated that nanowires dispersed in solution can be aligned on the substrate by electric field^1^,^2^, magnetic field^3^,^4^,^5^, fluid flow^6^,^7^,^8^, capillary force^9^,^10^,^11^,^12^,^33^,^34^, oil/water interface^13^,^14^ and others^15^,^16^,^17^,^18^,^19^,^20^,^21^,^22^,^35^. Among them, methods using lithographically defined patterns are promising for assembling heterogeneous nanowires due to the spatial controllability of patterns^10^,^11^,^12^,^33^,^35^. These methods require nanoscale patterns, which correspond to nanowire sizes. This requirement essentially limits the available size range in the size selectivity due to the size limitation of lithography^36^,^37^. Since one of major advantages for nanowires is the size range beyond the limitation of lithography, the positioning method for nanowires, whose size range is not limited by lithography, is highly desired.

This study proposes a size selective deposition technique and sequential alignment of nanowires by utilizing micrometer scale hydrophilic/hydrophobic patterned substrate. We utilized nanowires dispersed within oil, which were preferentially deposited only at a water/oil interface onto the hydrophilic patterns. We found the nanoscale size selectivity of nanowires deposited onto micrometer scale hydrophilic patterns. This nanoscale size selectivity by micrometer scale patterns can be extended to the sequential alignment of different sized nanowires on the same substrate.

## Results

**Figure 1:**
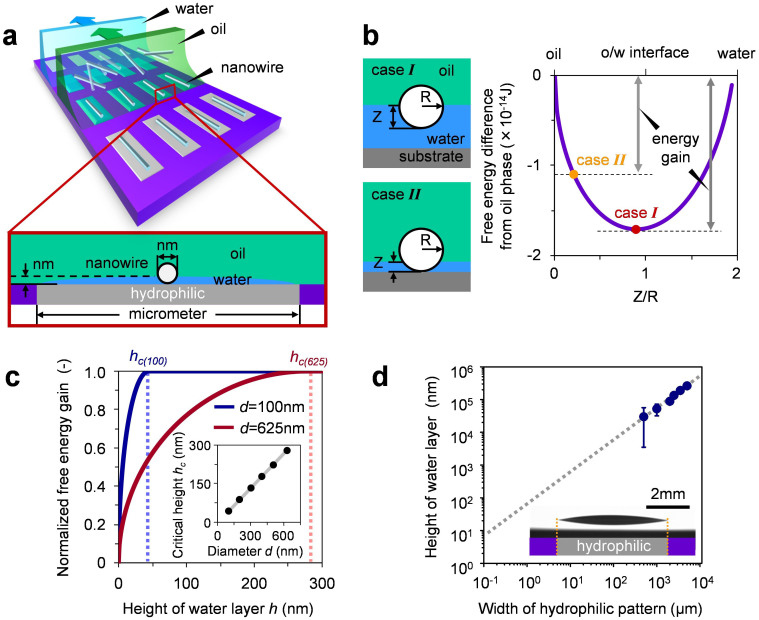
(a) Schematic illustration of nanoscale size selective deposition of nanowires utilizing micrometer hydrophilic patterns. (b) Effect of height of water layer on the nanowire depositions on the hydrophilic patterns. Schematic illustration and calculated free energy data of Si nanowire (100 nm diameter and 4.5 μm length) at oil/water interface were shown. The solid lines represent the possible free energy difference when the nanowire is adsorbed from oil to oil/water interface. Z is the distance between the oil/water interface and the nanowire bottom in the water. (c) Normalized free energy gain data as a function of height of water layer for nanowires with the diameters of 100 nm and 625 nm. The free energy values were normalized by the maximum value of free energy gain. The inset shows the critical height as a function of nanowire diameter. The critical height is defined as the height of water layer below which the free energy gain tends to decrease due to the geometrical limitation for the contact between nanowire and water. (d) Measured data of the height of water layers when varying the hydrophilic pattern width. The data was averaged from each 10 measurements. The inset shows the microscopy image of water droplets formed on the hydrophilic pattern.

Fig. 1a shows the schematic illustration of proposed method to exhibit the size selectivity of nanowires by utilizing micrometer scale hydrophilic/hydrophobic patterned substrate. In this method, water is first blade-coated onto the hydrophilic patterns of substrate. Before the water evaporates, Si nanowires dispersed within oil (1,4-dichlorobutane) is blade-coated onto the same substrate. During this coating process, the oil dispersion comes into contact with the water layer on the hydrophilic patterns, creating the water/oil interface. Nanowires dispersed within oil are preferentially adsorbed only at such water/oil interface to minimize Gibbs free energy of system^13^,^38^, as shown in the calculation data of Fig. 1b and Supplementary Information-S1. The key idea of the present method for the size selectivity of nanowires is to utilize the nanoscale height variation of water layers formed onto the micrometer scale hydrophilic patterns. As shown in Fig. 1b and c, our free energy calculations predict the emergence of size selectivity of deposited nanowires on micrometer scale hydrophilic patterns when the height of water layer is below around the radius of nanowires. This is due to the geometrical limitation for the contact between nanowire and water layer. Fig. 1c shows the calculated free energy gain data when varying the height of water layer. The free energy gain tends to decrease below the critical height of water layer h_c_, which is slightly lower than the radius of nanowires, as shown in the inset of Fig. 1 (c). Thus, the size selective deposition of nanowires onto the hydrophilic patterns might emerge if we can control the height of water layer at the size scale of nanowire radius. In general, the height of water layer formed on micrometer scale hydrophilic patterns is much lower than the width of patterns due to the wetting nature^39^,^40^,^41^. Our measurements for the height of water layers demonstrated that the height values can be varied at nanoscale range when the pattern width is below 10 μm, as shown in Fig. 1d and Supplementary Information-S2. Thus, it might be possible to exhibit the nanoscale size selectivity of nanowires by utilizing the nanoscale height variation of water layers formed onto the micrometer scale hydrophilic patterns.

**Figure 2:**
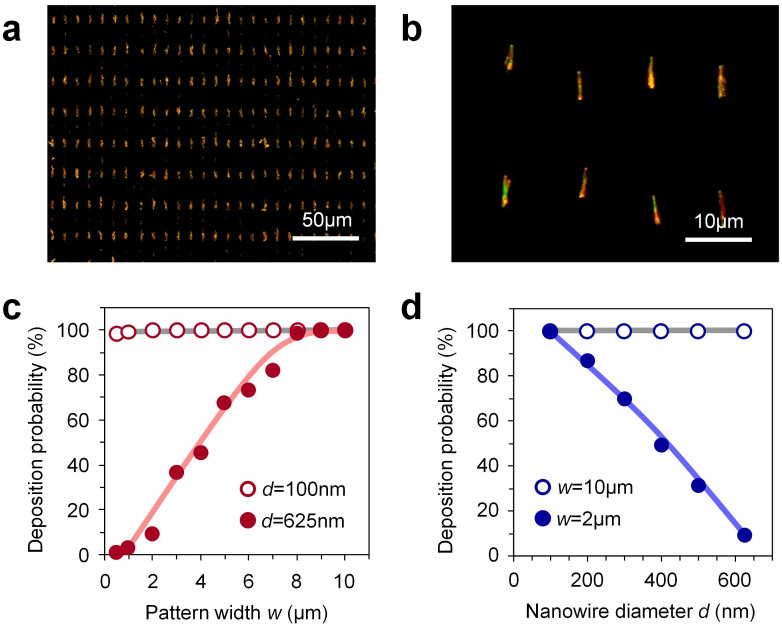
(a) Typical dark field optical microscopy and (b) magnified images of aligned 625 nm diameter nanowire on 3 μm width of hydrophilic patterns. (c) Deposition probability data of nanowires when varying hydrophilic pattern width. The data for nanowires with the diameters of 100 nm and 625 nm were shown. (d) Deposition probability data of nanowires when varying nanowire diameters. The data for the hydrophilic pattern widths of 2 μm and 10 μm were shown. The coating cycles are 20 for the experiments in Figure 2 (c) and (d).

Fig. 2a and b shows the typical dark field optical microscopy images of aligned Si nanowires. In this experiment, the nanowire diameter, the pattern size (width × length), the coating speed, the coating cycles and temperature were 625 nm, 3 μm × 10 μm, 10 mm/s, 100 and 20°C, respectively. The employed Si nanowires were fabricated by metal-assisted chemical etching method^42^,^43^,^44^. In Fig. 2a, the deposition probability of nanowires for a pattern was 98%. The deposition probability is defined as the number probability of patterns where the nanowire exists. Fig. 2c and 2d show the deposition probability of nanowires when varying the pattern width and the nanowire diameter. For the experiments in Fig. 2c, the nanowires with the diameter of 100 nm and 625 nm were used. As seen in Fig. 2c, the deposition probability of nanowires with the diameter of 625 nm decreased from 100% to 2.7% when decreasing the pattern width from 10 μm to 0.5 μm. On the other hand, the deposition probability of nanowires with the diameter of 100 nm was kept to be almost 100% even varying the width of patterns. For the experiments in Fig. 2d, the hydrophilic patterns with the widths of 2 μm and 10 μm were employed. When increasing the nanowire diameters from 100 nm to 625 nm for the constant width of pattern-2 μm, the deposition probability decreased from 100% to 9.3%, as shown in Fig. 2d. For the pattern width of 10 μm, the deposition probability of nanowires was almost 100% independent of the nanowire diameter. Thus these results of Fig. 2c and 2d clearly highlight the occurrence of the size selective deposition of nanowires using micrometer scale hydrophilic/hydrophobic patterned substrate.

**Figure 3:**
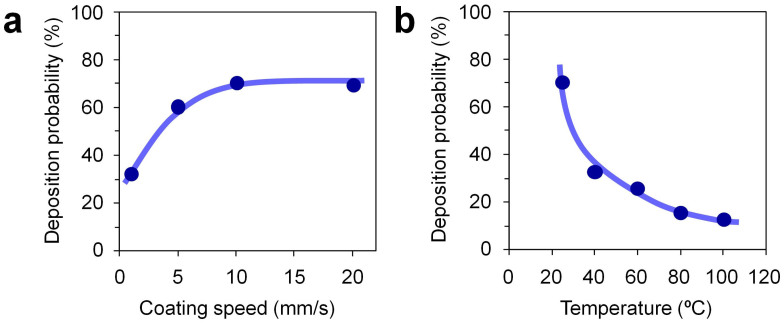
(a) Deposition probability data of nanowires when varying the blade coating speed at 20°C. (b) Deposition probability data of nanowires when varying the temperature for depositions with the blade-coating speed of 10 mm/s. In these deposition experiments, the nanowire diameter, the pattern size and the coating cycles were 300 nm, 2 μm × 10 μm and 20, respectively.

Next, we examine what really causes the size selective deposition in Fig. 2. If our present scenario based on the nanoscale height variation of water layer on the micrometer hydrophilic patterns is correct to explain the present results, the evaporation events of the water layer should play an important role on the observed nanowire depositions. To validate the scenario, we intentionally varied the height of water layer during deposition processes by varying the temperature and the blade-coating speed for depositions. Fig. 3a and 3b show the effects of temperatures and the blade-coating speeds on the deposition probability of nanowires. In these deposition experiments, the nanowires with the diameter of 300 nm and the hydrophilic pattern width of 2 μm were employed. When decreasing the blade-coating speed, the interval time between the water layer deposition and the oil layer deposition increases. The longer interval time must promote the evaporation of water layers before depositing oil dispersion with nanowires. As seen in Fig. 3a, decreasing the blade-coating speed resulted in the decrease of the deposition probability of nanowires. When increasing the deposition temperature, the deposition probability of nanowires decreased, as shown in Fig. 3b. This is clearly due to the enhancement of water layer evaporation via the increased deposition temperature. Thus, these two data demonstrate the critical role of water layer on the deposition probability of nanowires.

**Figure 4:**
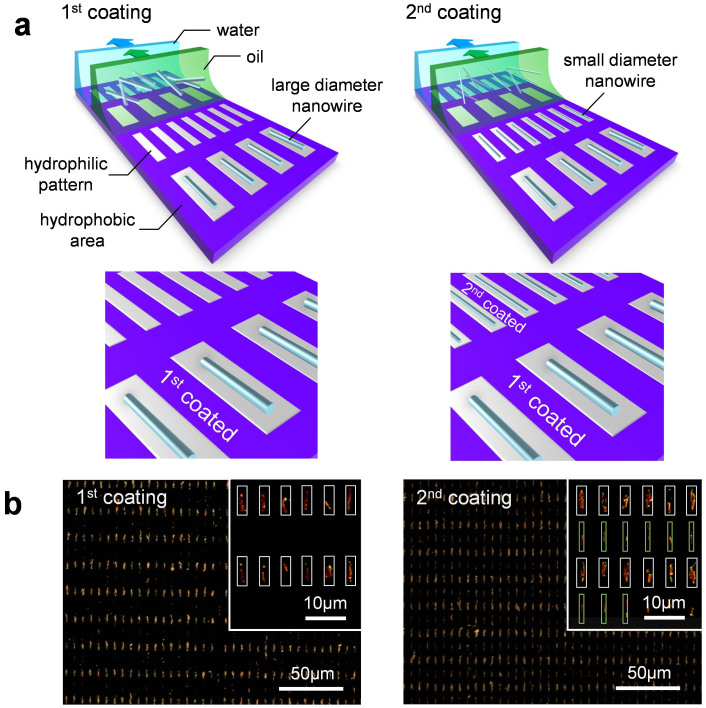
(a) Schematic illustration of sequential alignment of different sized nanowires. (b) Dark field optical microscopy images of nanowire alignment. Left image shows the data for 1^st^ blade-coating by nanowires with the diameter of 625 nm, and the right image shows the data for 2^nd^ blade-coating by nanowires with the diameter of 100 nm. The sizes (width × length) of large and small patterns were 3 μm × 8 μm and 500 nm × 8 μm, respectively. The coating cycles for 1^st^ and 2^nd^ blade-coatings were 100 and 10, respectively.

Based on above size selective deposition phenomenon using micrometer hydrophilic patterns, we demonstrate the sequential alignment of heterogeneous nanowires on the substrate. Fig. 4a shows the schematic illustration how the sequential alignment of different sized nanowires can be performed. Since relatively large sized nanowires cannot be deposited onto the shallow water layer on the hydrophilic patterns due to the above principle, sequential nanowire depositions for two different sized micrometer scale hydrophilic patterns might allow us to align selectively different sized nanowires only at the desired spatial locations. Here, we used 3 μm-width and 500 nm-width hydrophilic patterns. Two different diameter sized nanowires (625 nm and 100 nm) were sequentially deposited onto the patterned substrate. Fig. 4b shows the optical microscopy images of 1st deposition for nanowires of 625 nm diameter (left) and 2nd deposition for nanowires of 100 nm diameter (right). As clearly seen in the images, the two different sized nanowires were selectively deposited at each desired patterns. The deposition probabilities for the first and second depositions were > 97% and > 98%, respectively. Interestingly, during the 2nd deposition, the smaller nanowires were not deposited onto the patterns with predeposited larger nanowires. The existence of nanowires at the patterns seems to prevent the subsequent depositions of nanowires at the water/oil interface. Presumably, the repulsion force between nanowires and/or the decreased hydrophilicity due to the presence of nanowires might cause such phenomena. Thus, we successfully demonstrated the sequential alignment of different sized nanowires on the substrate by applying the nanoscale size selective phenomenon using micrometer hydrophilic patterns. Since the present size selective method utilizes the nanoscale height variation of water layer, the size range of selectivity could be further decreased by decreasing and controlling the height of water layer.

## Discussion

In summary, we report a size selective deposition and sequential alignment of nanowires by utilizing micrometer scale hydrophilic/hydrophobic patterned substrate. Nanowires dispersed within oil were preferentially deposited only at a water/oil interface onto the hydrophilic patterned area. The key idea to produce the nanoscale size selectivity is to utilize the nanoscale height variation of water layer naturally formed onto the micrometer hydrophilic patterns. We found that the deposited nanowire size was strongly limited by micrometer width of hydrophilic patterns. Thus it was possible to exhibit the nanoscale size selectivity of nanowires by utilizing micrometer scale hydrophilic patterns. Furthermore, we successfully demonstrated the sequential alignment of different sized nanowires on the same substrate by applying this size selectively phenomena.

## Methods

### Si Nanowires Fabrication

Si nanowires with the diameter ranged from 100 nm to 625 nm were fabricated by metal-assisted chemical etching method using Au mesh patterns on Si substrate. See the fabrication details in Supplementary Information-S3. The length of Si nanowires was controlled to be 4.5 μm.

### Si Nanowires Surface Modification

Si nanowire surfaces were chemically modified by 1% chloromethyl(trichloro)silane in a chloroform and hexadecane (1:4) solution for 2 hours at room temperature. These chemically modified Si nanowires were dispersed into 1,4-dichlorobutane by sonication. The concentration of nanowires was estimated to be 5.68 × 10^6^/μl.

### Hydrophilic/Hydrophobic Patterned Substrate Fabrication

The hydrophilic/hydrophobic patterns on the Si_3_N_4_/Si substrate were fabricated by conventional photolithography. The patterned substrates were immersed into 1% 1H,1H,2H,2H-perfluorooctyl(trichloro)silane (FOTS) in perfluorooctane for 2 hours at room temperature. After forming FOTS self-assemble layer on the substrate, the photoresist patterns were removed by ultra-sonication in acetone, N,N-dimethylformamide and isopropanol. The widths of patterns were ranged from 0.5 to 10 μm. 4 × 4 cm patterned substrate was used for blade-coating process.

### Blade Coating Alignment Process

The blade coating was performed by using two blades for Milli-Q water and 1,4-dichlorobutane with nanowires, sequentially. The distance between two blades was set to be 2 mm. During the blade-coating process, water was selectively deposited onto the hydrophilic patterns. 1,4-dichlorobutane with nanowires was subsequently deposited onto the water layer formed on the hydrophilic patterns.

## Author Contributions

Y.H., K.N., M.K. and T.Y. designed this work and prepared the manuscript. The experiments were carried out by Y.H., K.N., G.M., F.Z. and Y.H., K.N., M.K. G.M., F.Z., S.R. X.L., T.K., T.Y. have analyzed the results and discussed the manuscript during the preparation. All authors discussed the results and implications and commented on the manuscript at all stages.

## Supplementary Material

Supplementary Information Supplementary Information
