# Supplementary material for: Nanoscale Size-Selective Deposition of Nanowires by Micrometer Scale Hydrophilic Patterns
Source: Sci Rep. 2014 Aug 4;4:5943. doi: 10.1038/srep05943 (PMC4120308; doi:10.1038/srep05943)
Supplement: Supplementary Information [file srep05943-s1.pdf]

Supplementary information

Nanoscale Size-Selective Deposition of Nanowires by Micrometer Scale Hydrophilic Patterns

Yong He,[1] Kazuki Nagashima,[1] Masaki Kanai,[1] Gang Meng,[1] Fuwei Zhuge,[1] Sakon Rahong,[1] Xiaomin Li,[2] Tomoji Kawai [1] and Takeshi Yanagida [1]

[1]The Institute of Scientific and Industrial Research, Osaka University, 8-1 Mihogaoka Ibaraki, Osaka, 567-0047, Japan

[2] Shanghai Institute of Ceramics, Chinese Academy of Sciences, Shanghai, 200050, China

S1. Gibbs free energy calculation for the present systems

Here we describe the details of free energy calculations for the total energy change of nanowire from oil to oil/water interface: [1, 2] The free energy gain can be calculated by using the following formula:

$$\Delta G=LA(\gamma_{nw}-\gamma_{no})-LC\gamma_{ow}$$

where L, A, and C are the nanowire length, arc length (nanowire/water interface) and chord length (water/oil/nanowire interface) of nanowire cross section, respectively. The values of the interfacial tensions between the nanowire and water γ_{nw}, the interfacial tensions between the nanowire and oil γ_{no}, and the interfacial tensions between the oil and water γ_{ow} are 11.4 mJ/m^2, 6.7 mJ/m^2, and 45.0 mJ/m^2, respectively, based on the previous reports [1].

S2. Measurements of water layers on hydrophilic patterns

The water layer formed onto the hydrophilic patterns plays an important role on the nanowire deposition phenomena in the present study. Although the direct measurement of the water layer is ideal to reveal the role, it is difficult to directly measure the nanoscale height of water layer due to the evaporation event during the measurements. For example, as reported by A. J. Petsi and V. N. Burganos, [1] the change of water layer height for the water droplet with fixed contact line can be expressed as:

$$dh_0/dt=-(J_0/\rho)(\theta_c-\theta_c cos\theta_c)/(sin\theta_c-\theta_c cos\theta_c)$$

where h_0 is the height of water layer, t is time, J_0 is the evaporation flux, ρ is the water density and θ_c is the contact angle. Assuming the constant contact angle, the change of water layer height by evaporation event is constant and independent on the water droplet size. If the change of water layer height is several tens nm scale, the contribution of water evaporation is negligible for sub-millimeter water thickness but involves serious error for tens to hundreds nanometer water layer height. In fact, the error bars for the height of water layer became larger as the hydrophilic pattern width decreased. To minimize the contribution of water evaporation, we conducted the measurement only at millimeters scaled pattern. Fig. SI shows the measured water layer height distribution data as a function of hydrophilic pattern width. The water layer height was defined as the height data at the middle top of water layer on the hydrophilic patterns. The relationship between the water layer height and the hydrophilic pattern width was found to be liner on the log-log plots, as seen in Fig. SI. This proportional relationship between the water droplet size and the height of water layer is theoretically supported by Ref. 3 as following equations:

$$h_0=R_b(1-\cos\theta_c)/\sin\theta_c$$

where R_b is the half-width of the wetted area. In addition, we found the good consistency between the estimated height of water layer in figure 1d and the experimental results of nanowire alignment in figure 2d. Specifically, the deposition probability for 10 μm hydrophilic pattern with the estimated height of water layer of ca. 500 nm was kept almost 100 % in the nanowire diameter range of 100-625 nm, while that for 2 μm hydrophilic pattern with the estimated height of water layer of ca. 100 nm

decreased when the nanowire diameter was over 200 nm. These data strongly supports that our estimation of the height of water layer is qualitatively collect.

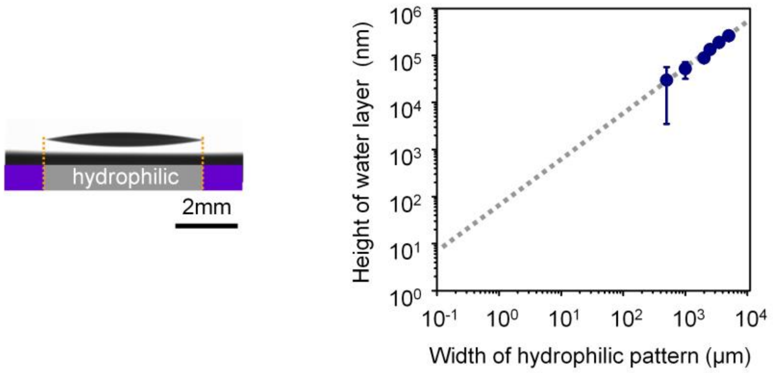

Fig. SI The microscopy image of water droplets formed on the hydrophilic pattern and measured data of water layer heights on the hydrophilic patterns. The data was averaged from each 10 measurements.

S3. Fabrication and characterization details of Si nanowire arrays

Si nanowires with the diameters ranged from 100 to 625 nm were fabricated by metal-assisted chemical etching method [4, 5]. The schematic illustration can be seen in Fig. SIIa. The diameter size of Si nanowires was defined by controlling Au mesh pattern sizes on the silicon substrate. First, photoresist (AZ5206:AZ5200=2:1) was coated on p-type Si substrate (resistivity = 0.01-0.02 Ω) by spin-coating with the rotation speed of 5500 rpm for 60 sec, and subsequently the coated substrate was baked at 90 °C for 3 min. The array patterns ranged from 100-625 nm were fabricated by performing nano-imprint lithography on the resist-coated substrate. The pattern sizes were defined by pattern sizes of polydimethylsiloxane (PDMS) molds, which were made via Si master mold [4, 6]. After performing nano-imprint lithography, the

patterned photoresist layer was solidified at 120 °C for 5 min. Then, Au metal layer with the thickness of 30 nm was deposited onto the patterned substrate by using RF sputtering. The residual photoresist were removed by sonication within N,N-dimethylformamide solution for 30 min. The fabricated patterned substrate was immersed into an etchant solution of 4.60 M hydrofluoric (HF) and 0.44 M hydrogen peroxide (H_2O_2). This etching process time was controlled to achieve the desired length of Si nanowires. Then the etched substrates were rinsed by Milli-Q water.

The scanning electron microscope (SEM) images of Au patterns and the fabricated Si nanowires with the diameter of 200 nm were shown in Fig. SIIb and S1c. Fig. SIId shows the TEM image of fabricated Si nanowires. The selected area diffraction pattern was shown in the inset. The TEM and diffraction data demonstrated that the fabricated Si nanowires were single crystals. The length of Si nanowires was controlled to be about 4.5 μm by controlling the etching time. The surface of fabricated Si nanowires was then chemically modified by 1% chloromethyl(trichloro)silane in a chloroform and hexadecane (1:4) solution for 2 hours at room temperature. The chemically modified Si nanowires could be well dispersed within 1, 4-dichlorobutane by ultra-sonication, as shown in the inset of Fig. SIIe. Fig. SIIe shows the length distribution of Si nanowires in solvent after ultra-sonication process to remove the nanowires from the substrate. As can be seen, the length data for 80.4 % of all Si nanowires were within the range between 4 and 5.5 μm.

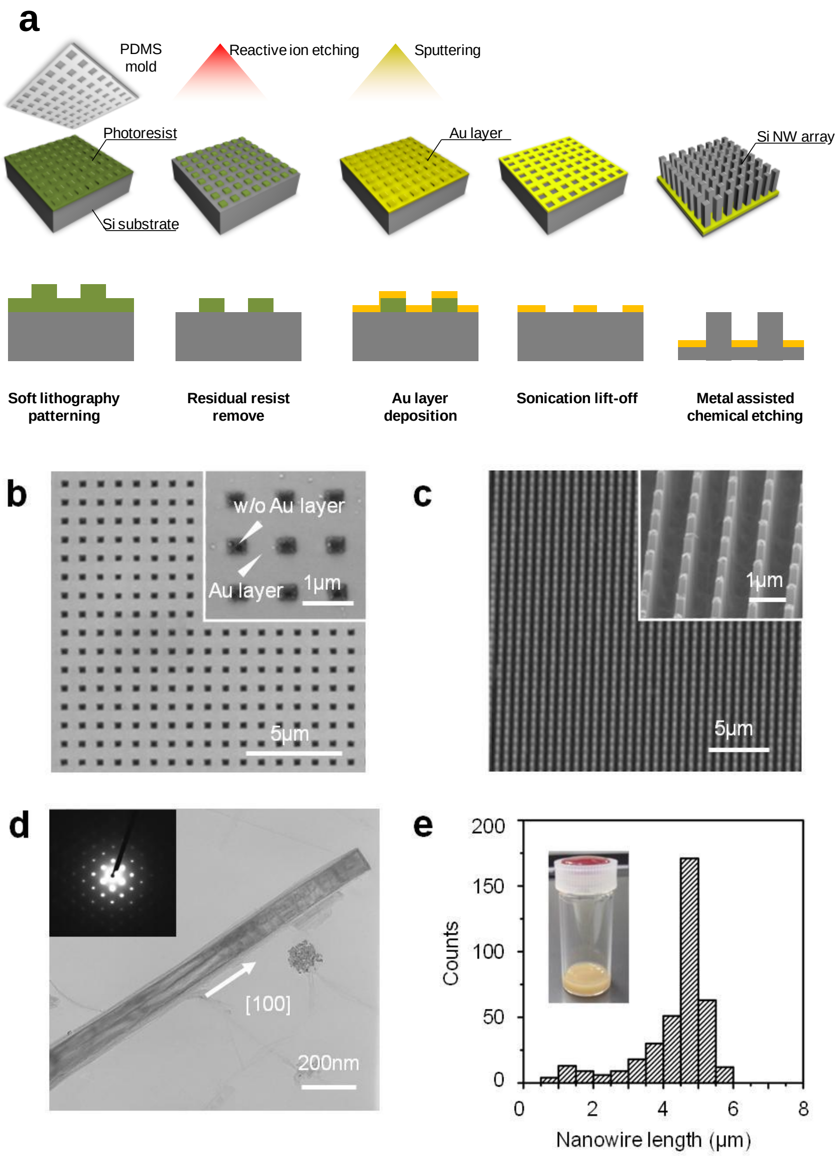

Fig. SII (a) Schematic illustration of fabrication of Si nanowire arrays. (b) SEM images of fabricated patterned Au layer on Si substrate. (c) SEM images of Si nanowires. (d) TEM images and selective area electron diffraction (SAED) pattern (inset) of Si nanowires. (e) Length distribution of Si nanowires dispersed in 1, 4-dichlorobutane.

S4. Nanowire deposition probability when varying blade-coating cycles

The effect of blade-coating cycles on the nanowire deposition probability was shown in Fig. SIIIa. When blade-coating cycles increased, the nanowire deposition probability increased from 12 % to 98 %, as shown in Fig. SIIIb. In these experiments, the nanowire diameter, the pattern size, the coating speed and temperature were set to be 625 nm, 3 μm x 10 μm, 10 mm/s and 20 °C, respectively.

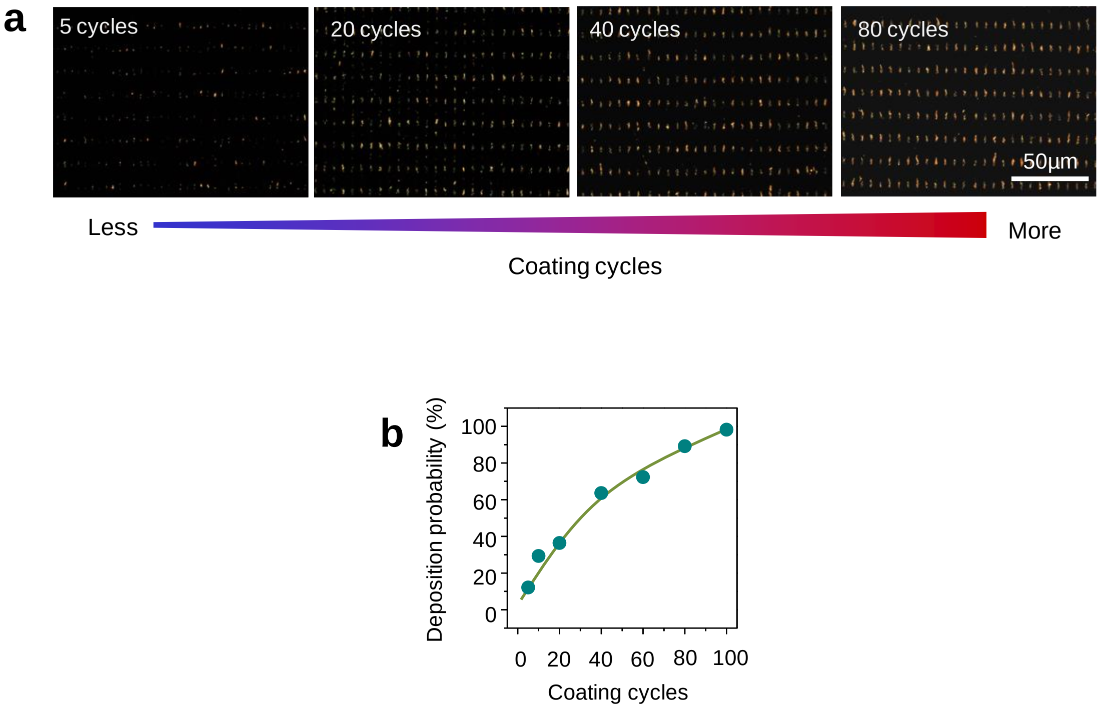

Fig. SIII (a) Dark field optical microscopy images of aligned Si nanowires when varying blade-coating cycles. (b) Nanowire deposition probability data as a function of blade-coating cycles.

S5. Deposition probability and the number of nanowires within a single pattern when varying pattern width

Fig. SIVa and SIVb show the optical microscopy images of aligned Si nanowires of the diameter of 200 nm on the hydrophilic patterns with the widths of 3 μm and 1 μm. Although the nanowire deposition probability decreased from 90.5 % to 71.8 % when the hydrophilic pattern width decreased from 3 μm and 1 μm, the percentage of single nanowire deposition per a single pattern increased from 46.3 % to 68.5 %, as shown in Fig. IV4c. The number of nanowires per pattern was measured for 700 patterns and the blade-coating in these experiments was performed for five cycles.

Although our theory does not predict that only a single nanowire is deposited onto each pattern, a single nanowire deposition can be achieved by precisely controlling the height of water layer. As shown in Figure SVb and SVd, when the 5 μm hydrophilic pattern was used for nanowire alignment, the height of water layer was large enough, therefore many nanowires could be deposited onto each hydrophilic pattern. When the height of water layer decreased, the number of nanowire per pattern reduced, as shown in Figure SVa and SVc. In principle, more perfect single nanowire alignment can be achieved by precisely controlling the height of water layer. It can be modulated not only by the pattern size, but also by the environmental temperature and humidity.

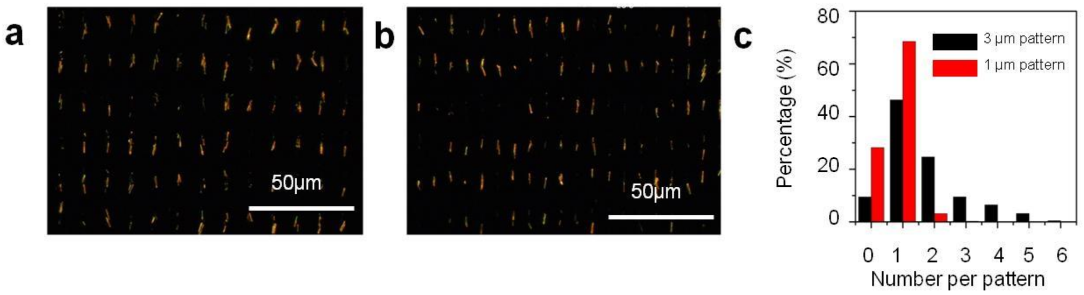

Fig. SIV Dark field optical microscopy images of aligned Si nanowire on (a) 3 μm and (b) 1 μm width patterns. (c) The distribution data of numbers of deposited nanowires per a pattern.

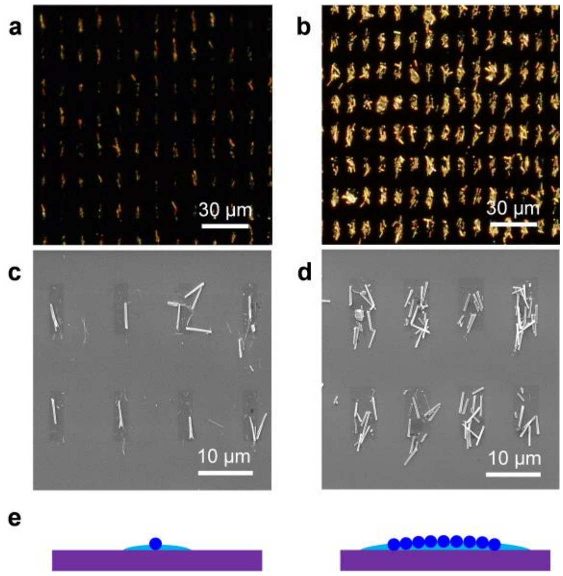

Fig. SV (a,b) Dark field optical microscopy images and (c,d) SEM images of aligned Si nanowire on (a,c) 2 μm and (b,d) 5 μm width patterns. (e) Schematic of nanowire alignment on different pattern width.

References

1. Nakagawa, T., Torii, H., Kawashima, T. & Saitoh, T. Controlled deposition of silicon nanowires on chemically patterned substrate by capillary force using a blade-coating method. *J. Phys. Chem. C* **112**, 5390-5396 (2008).
2. Hu, L. F., Chen, M., Fang, X. S. & Wu, L. M. Oil-water interfacial self-assembly: a novel strategy for nanofilm and nanodevice fabrication. *Chem. Soc. Rev.* **41**, 1350-1362 (2012).
3. A. J. Petsi & V. N. Burganos. Potential flow inside an evaporating cylindrical line. *Phys. Rev. E* **72**, 047301 (2005).

4. Huang, Z., Geyer, N., Werner, P., de Boor, J. & Goesele, U. Metal-Assisted Chemical Etching of Silicon: A Review. *Adv. Mater.* **23**, 285-308 (2011).
5. Geyer, N. et al. Model for the Mass Transport during Metal-Assisted Chemical Etching with Contiguous Metal Films As Catalysts. *J. Phys. Chem. C* **116**, 13446-13451 (2012).
6. He, Y. et al. Crystal-Plane Dependence of Critical Concentration for Nucleation on Hydrothermal ZnO Nanowires. *J. Phys. Chem. C* **117**, 1197-1203 (2013).
